# A validation study to analyze the reliability of center of pressure data in static posturography in dogs

**DOI:** 10.3389/fvets.2024.1353824

**Published:** 2024-03-14

**Authors:** Masoud Aghapour, Nadja Affenzeller, Christiane Lutonsky, Christian Peham, Alexander Tichy, Barbara Bockstahler

**Affiliations:** ^1^Section of Physical Therapy, Small Animal Surgery, Department for Companion Animals and Horses, University of Veterinary Medicine, Vienna, Austria; ^2^Clinical Unit of Internal Medicine Small Animals, Department for Companion Animals and Horses, University of Veterinary Medicine, Vienna, Austria; ^3^Movement Science Group, Equine Surgery, Department for Companion Animals and Horses, University of Veterinary Medicine, Vienna, Austria; ^4^Platform Bioinformatics and Biostatistics, Department for Biomedical Services, University of Veterinary Medicine, Vienna, Austria

**Keywords:** kinetics, gait analysis, COP, ground reaction forces, inter-observer reliability, test-retest reliability, dog

## Abstract

**Introduction:**

Center of pressure (COP) parameters are frequently assessed to analyze movement disorders in humans and animals. Methodological discrepancies are a major concern when evaluating conflicting study results. This study aimed to assess the inter-observer reliability and test-retest reliability of body COP parameters including mediolateral and craniocaudal sway, total length, average speed and support surface in healthy dogs during quiet standing on a pressure plate. Additionally, it sought to determine the minimum number of trials and the shortest duration necessary for accurate COP assessment.

**Materials and methods:**

Twelve clinically healthy dogs underwent three repeated trials, which were analyzed by three independent observers to evaluate inter-observer reliability. Test-retest reliability was assessed across the three trials per dog, each lasting 20 seconds (s). Selected 20 s measurements were analyzed in six different ways: 1 × 20 s, 1 × 15 s, 2 × 10 s, 4 × 5 s, 10 × 2 s, and 20 × 1 s.

**Results:**

Results demonstrated excellent inter-observer reliability (ICC ≥ 0.93) for all COP parameters. However, only 5 s, 10 s, and 15 s measurements achieved the reliability threshold (ICC ≥ 0.60) for all evaluated parameters.

**Discussion:**

The shortest repeatable durations were obtained from either two 5 s measurements or a single 10 s measurement. Most importantly, statistically significant differences were observed between the different measurement durations, which underlines the need to standardize measurement times in COP analysis. The results of this study aid scientists in implementing standardized methods, thereby easing comparisons across studies and enhancing the reliability and validity of research findings in veterinary medicine.

## 1 Introduction

Gait analysis is the most clinically relevant tool in veterinary medicine to study locomotion and diagnose movement disorders in animals such as lameness (1–3). Evaluation of ground reaction forces (GRF) of a participant while walking or trotting over a force (4, 5) or pressure plate (1, 3) is considered the gold standard for objective lameness diagnosis (2, 6, 7). In recent years these systems have not only been used to detect lameness in a more quantifiable and reliable way but also to study postural balance in different species (2).

Postural balance is based on the integration of sensory input from the proprioceptive, visual, and vestibular systems alongside adaptive muscular activations coordinated within the central nervous system (8, 9). Balance itself is maintained by muscle contractions, acting against gravity, and forms the basis of many daily activities such as walking and standing. Hence, standing still is an active, controlled process, influenced by the aforementioned sensorimotor signals. Impairments in any of these systems can negatively affect postural stability in humans (10, 11). Therefore, studying the postural balance provides valuable information about the interactions between these systems.

One important method to study postural stability during walk and trot (dynamic posturography) or quiet standing (static posturography) is the center of pressure (COP) of the body and/or each limb. Even when standing still, the body is constantly moving in the craniocaudal (anterior-posterior in human) and mediolateral directions. Therefore, the center of gravity of the body and center of mass (COM) continue to move to keep the COM within the base of support to prevent falling over. Controlled muscular contractions change the repartition of force under the body resulting in continuous fluctuations of the COP with the goal to keep the COM in a fairly constant position. Consequently, the COP under the feet or paws permanently shifts (12, 13). The COP is the point at which the instantaneous vector of the GRF is applied (12) so that the displacement of the COP is an indirect measure of the functionality of postural stability and thus a measure of the ability to maintain balance. The performance of postural control is usually assessed by quantifying COP-based measures of postural sway during a quiet stance (14). Thus, for example, lower COP values are assumed for better stability in static posturography (12, 15).

Not surprisingly, scientists in veterinary medicine used COP parameters not only to study the influence of different physical demands on clinically healthy dogs (16, 17) but also to assess the effect of different orthopedic disorders such as hip dysplasia (18, 19), elbow dysplasia (13, 15, 19, 20), cranial cruciate ligament rupture (15), and different neurologic disorders such as thoracolumbar spinal cord injuries (21, 22). Recently, static posturography has been used to assess body weight distributions, left-right symmetry indices, and the effect of breed and age (23–25).

In view of these highly relevant research questions, the methods already published by the various authors vary tremendously. The analysis of GRF (23, 26–32) and COP (2, 13, 15, 20, 25, 33–46) during static measurements on pressure and force plates has been performed in numerous veterinary publications. Despite following a common principle where animals were motivated to stand still for a predetermined period without movements of the head and the feet, a wide variety of techniques, especially regarding the duration and number of trials, is seen in the literature. The measurement duration ranges from 30 s (34) to 1 s (25, 41) with the most common choice for a trial duration in assessing GRF (26, 27, 30) and COP in both dogs (20, 35, 37) and horses (39, 42, 44) being a 10 s period per trial. Likewise, the number of repetitions varies from 1 (25, 41) to 8 (33, 45), while some authors state a range or a minimum of repetitions (23, 33, 37, 41, 44). The most frequently used combinations for COP measurement in dogs are continuous periods of 20 s (13, 15) and 10 s (20, 35) repeated three times (for more information please see overview in Supplementary Table 1). However, no scientific consensus, especially regarding minimum measurement duration and the minimum number of trials in static posturography has been published so far.

This variability in methodology potentially impacts reported results, making it difficult to accurately compare findings across different studies and different species. However, identifying and evaluating potential confounding factors is crucial for obtaining reliable and robust COP results. This is why, in this study, we aimed to assess the inter-observer reliability and test-retest reliability of body COP parameters of dogs during quiet standing (static posturography) on a pressure plate. Furthermore, adhering to the 3R principle (Replacement, Reduction, and Refinement), our goal was to determine the minimum number of trials and the shortest duration necessary to ensure a high level of accuracy in the evaluated COP data. We believe that this aligns with the principles of refinement in animal trials (47).

## 2 Materials and methods

### 2.1 Approval and consent

This study was approved by the Ethics and Animal Welfare Committee of the University of Veterinary Medicine, Vienna in accordance with the University's guidelines for Good Scientific Practice guidelines and national legislation (ETK-148/10/2021).

### 2.2 Animals

A total of 26 client-owned dogs were evaluated in this study. The inclusion criteria consisted of the absence of any clinical musculoskeletal, neurological, or visual disease; thus, all dogs underwent a general clinical examination by qualified veterinarians (MA, NA, CL) including visual gait assessment, joint palpation, and calculation of the symmetry index (SI). One dog was excluded from the study due to abnormal clinical examination findings, and an additional 13 dogs were excluded due to the inability to stand still for a minimum of 30 s. As a result, 12 healthy dogs were included in this study. The breed of the dogs consisted of Labrador retriever (*n* = 4), border collie (*n* = 2), standard poodle, Flat-Coated retriever, Malinois, Irish terrier, pointer, and 1 mixed breed dog. The body weight and age of the dogs ranged from 32.5 to 13.5 kg (22.68 ± 5.43) and 6.0 to 1.11 years (3.39 ± 1.79), respectively. Five dogs were male (all intact), and seven dogs were female (five intact and two spayed). All dogs had to weigh more than 10 kg to be included in the study.

### 2.3 Equipment and measurement procedure

In this prospective study, the COP parameters of the dogs were measured in a quiet standing position on a flat ground by using a Zebris platform (FDM Type 2, Zebris Medical GmbH, Allgäu, Germany) equipped with 15,360 sensors covering an area of 203 × 54.2 cm and a measuring frequency of 100 Hz. The sensor size of the platforms was 0.72 × 0.72 cm. To standardize the coefficient of friction, the pressure plate was covered with a black 1-mm-thick non-slip rubber mat made of polyvinyl chloride. All measurement procedures were filmed using a Panasonic NV-MX500 camera (Panasonic, Kadoma, Osaka, Japan) with a standardized setup for camera positioning and angle. The camera was placed 3.5 m away from the origin (coordinate 0,0) of the platform at an angle of 35° to the long side of the platform, to capture both sagittal and frontal views. It was set at a height of 0.85 m with a 90° angle to ensure comprehensive coverage of the observed area (Supplementary Figure 1).

In this study, the assessment of the SI during the walk and posturography of the included dogs was conducted by different observers (MA, NA, or CL). Once the SI was confirmed to fall within an acceptable range (SI < 3%), posturographies, consisting of three trials (M1–M3) for each dog, were recorded by one of the aforementioned observers. Subsequently, each of the three observers independently analyzed the recorded trial data and defined the starting point for the 20 s measurement duration for each dog separately.

#### 2.3.1 Evaluation of symmetry index during walk

In order to familiarize the dogs with the room, the set-up, and the equipment, they were allowed to wander around freely for up to 15 min. Once the dogs were familiarized with the room, GRF was measured when walking in a straight line using the pressure plate mentioned above. At least five valid passes were analyzed to calculate the SI for peak vertical force (PFz) and vertical impulse (IFz) as reported in the literature (17, 19, 48). A valid pass was defined as a walk in which the dog crossed the plate without changing pace, turning its head, pulling on the lead, or touching the dog handler (MA, NA, or CL). The difference in speed at which the dogs crossed the plate had to be within a range of ± 0.3 m/s and an acceleration of ± 0.5 m/s^2^ (7, 49, 50). The SI for PFz, and IFz of all included dogs in this study was below 3% which is the margin typically used to distinguish between a sound and asymmetric gait pattern (17, 19, 48).

#### 2.3.2 Posturography

After a short break, COP parameters (see details in Section 2.4) were measured for each dog. The dogs had to stand still on the pressure platform with all limbs perpendicular to the platform. Each trial lasted for a minimum of 30 s and was repeated three times (M1, M2, and M3) for each dog. The dogs were able to have a break between trials and received treats after each trial. Data were discarded by the observer if any body, head, tail, limb, or paw movements were observed on the video recording.

### 2.4 Data analysis

The following parameters were used for the evaluation of the inclusion criteria:

- The mean speed (m/s) and acceleration (m/s^2^) were calculated for the left frontlimb using the pressure plate data. For that purpose, two consecutive paw impacts (beginning of touch down) on the plate were used. The following formulas were used:
  ° Step length (distance in m) = the difference between the center of paw contact area of the second and first paw impact (plate coordinates)
  ° Step duration (s) = time between two consecutive paw impacts
  ° $Speed\;\left(m/s\right)\;=\;\frac{step\;length}{step\;duration}$
  ° Acceleration (m/s^2^) = speed of the second step – speed of the first step
- Symmetry index (SI) expressed as a percentage (SI%), was calculated for both parameters (PFz and IFz) according to the following equation modified from Budsberg et al. (48):

$$\begin{array}{l} SIXFz\;\left(\%\right)=abs\;\left(\frac{\left[\;XFzLLx\;-\;XFzRLx\;\right]}{\left[XFzLLx\;+\;XFzRLx\right]}\right)\times \;100 \end{array}$$

Where XFz is the mean value of peak vertical force (PFz) or vertical impulse (IFz) of valid steps, LLx is the left front- or hindlimb, and RLx is the right front- or hindlimb; perfect symmetry between the right and left front- or hindlimbs was assigned a value of 0%.

Each of the three observers conducted separate analysis of the recorded trial data. Within each trial, 20 consecutive seconds were used for analysis, chosen independently by each observer (labeled as M1, M2, and M3). In dogs that did not stand still for 20 s, the longest valid period was chosen for analysis (for details see Table 1). Eight dogs completed all three trials instantaneously. For three dogs, one additional trial was needed to achieve three valid trials. One dog required two additional trials (in total five trials). Measured data were analyzed with the custom-made software Pressure Analyzer (Michael Schwanda, version 4.8.7.0). In the next step, every 20 s long sequence was divided into six categories by each observer independently for each valid trial:

- Category A: 1 sequence lasting 20 s (1 × 20 s).
- Category B: 1 sequence from the same selected 20 s lasting 15 s (1 × 15 s).
- Category C: 2 sequences from the same selected 20 s each lasting 10 s (2 × 10 s).
- Category D: 4 sequences from the same selected 20 s each lasting 5 s (4 × 5 s).
- Category E: 10 sequences from the same selected 20 s each lasting 2 s (10 × 2 s).
- Category F: 20 sequences from the same selected 20 s each lasting 1 s (20 × 1 s).

**Table 1:** Overview of incomplete categories per dog analyzed by each observer.

**Dog number**	**Observer**	**Trial**	**Maximum number of analyzed seconds in each category (A–F) for incomplete measurements**					
			**A**	**B**	**C**	**D**	**E**	**F**
			**1** × **20 s**	**1** × **15 s**	**2** × **10 s**	**4** × **5 s**	**10** × **2 s**	**20** × **1 s**
1	3	M2	n.p.	1 × 15 s^*^	1 × 10 s	3 × 5 s	7 × 2 s	15 × 1 s
4	1	M1	n.p.	1 × 15 s^*^	1 × 10 s	3 × 5 s	8 × 2 s	16 × 1 s
4	2	M1	n.p.	1 × 15 s^*^	1 × 10 s	3 × 5 s	8 × 2 s	17 × 1 s
4	3	M1	n.p.	1 × 15 s^*^	1 × 10 s	3 × 5 s	7 × 2 s	15 × 1 s
8	1	M2	n.p.	1 × 15 s^*^	1 × 10 s	3 × 5 s	8 × 2 s	16 × 1 s
8	1	M3	n.p.	n.p.	n.p.	n.p.	n.p.	n.p.
8	2	M3	n.p.	n.p.	n.p.	n.p.	n.p.	n.p.
8	3	M1	n.p.	1 × 15 s^*^	1 × 10 s	3 × 5 s	7 × 2 s	15 × 1 s
8	3	M3	n.p.	1 × 15 s^*^	1 × 10 s	3 × 5 s	7 × 2 s	15 × 1 s
9	1	M2	n.p.	1 × 15 s^*^	1 × 10 s	3 × 5 s	7 × 2 s	15 × 1 s
9	3	M2	n.p.	n.p.	1 × 10 s	2 × 5 s	5 × 2 s	10 × 1 s
10	1	M1	n.p.	n.p.	1 × 10 s	2 × 5 s	6 × 2 s	13 × 1 s
10	2	M1	n.p.	n.p.	1 × 10 s	2 × 5 s	7 × 2 s	14 × 1 s
10	3	M1	n.p.	n.p.	1 × 10 s	2 × 5 s	5 × 2 s	10 × 1 s
11	1	M3	n.p.	n.p.	n.p.	2 × 5 s	5 × 2 s	10 × 1 s
11	3	M3	n.p.	n.p.	n.p.	1 × 5 s	2 × 2 s	5 × 1 s
12	1	M1	n.p.	n.p.	1 × 10 s	2 × 5 s	6 × 2 s	12 × 1 s
12	2	M1	n.p.	1 × 15 s^*^	1 × 10 s	3 × 5 s	7 × 2 s	14 × 1 s
12	2	M2	n.p.	1 × 15 s^*^	1 × 10 s	3 × 5 s	8 × 2 s	18 × 1 s
12	3	M1	n.p.	1 × 15 s^*^	1 × 10 s	3 × 5 s	7 × 2 s	15 × 1 s
12	3	M2	n.p.	1 × 15 s^*^	1 × 10 s	3 × 5 s	7 × 2 s	15 × 1 s

n.p., not possible.

^*^Complete analyzed category.

All data from each dog and trial were exported as Excel files (Microsoft Excel 2016) for further analysis.

Calculation of the body COP:

The pressure sensors are distributed across the surface of the plate. Under load (exerted by the limbs of the dog) each sensor measures the pressure at its specific location. Based on the pressure measurements from all sensors, the pressure distribution across the entire plate can be calculated. The COP is then calculated by using the weighted averages of the pressure measurements, considering the location of each sensor and the measured pressure. The following formulas are used:


$$\begin{array}{l} COPx\;=\frac{\sum_{i}\left(P_{i}\cdot x_{i}\right)}{\sum P_{i}} \end{array}$$

$$\begin{array}{l} COPy\;=\frac{\sum_{i}\left(P_{i}\cdot y_{i}\right)}{\sum P_{i}} \end{array}$$

In this study, the following COP parameters (body COP) were calculated:

- Mediolateral displacement (MLD) in millimeters (mm).
- Craniocaudal displacement (CCD) in mm.
- Total length (L) in meters (m); defined as the length of the line connecting the coordinates of the COP trajectory at each timeframe.
- Average speed (AS) in mm per second; defined as the mean speed of the COP movement.
- Support surface (SS) in mm^2^; area of the ellipse containing 90% of all COP points.

**Figure 1:**
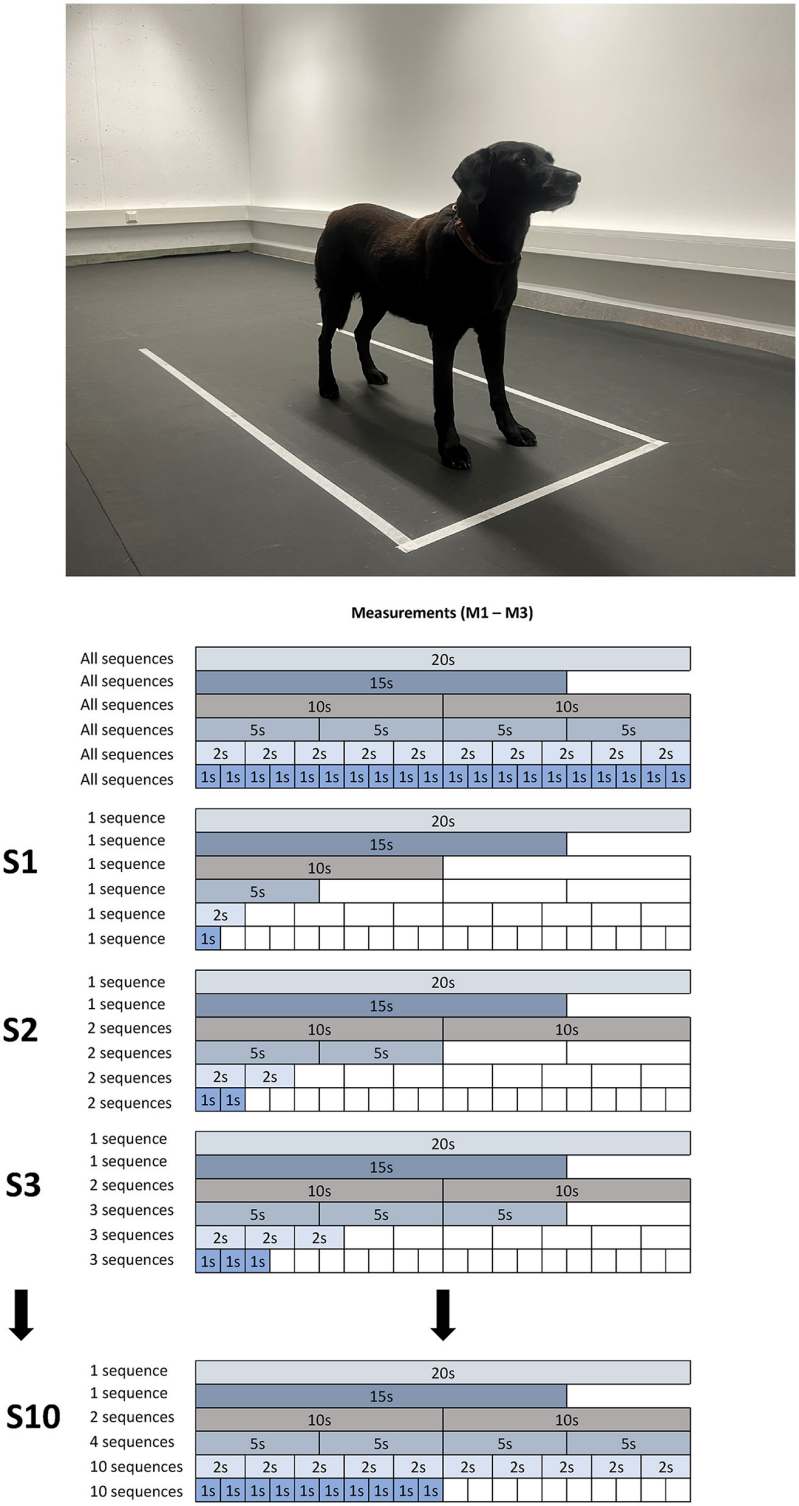
Representation of the selection of sequences to be examined using the example of Dog 11: initially, each investigator chose a 20 s sequence for each measurement (1–3). Afterward, the first 15 s were selected, followed by two sets of 10 s, four sets of 5 s, and so on. This served as the basis for the subsequent stepwise selection of sequences: initially, the respective 1st sequence was used, in the next step, the first two, then the first three, and so forth. The process concluded when 10 sequences of 1 s each were reached.

All analyzed data were sorted by number of dogs (1–12), observer (1–3), trial number per dog (M1–M3), category, and duration of each sequence (from 1 to 20 s). As a next step, from each category, 1–10 sequences (S1–S10) were selected for each COP parameter (for details see Figure 1).

In brief, S1 included the first measurement of category A-F, whereas S2 included the calculated mean of the first 2 measurements of every category (note that categories A and B only consisted of 1 dataset each in S2 and from thereon). This simultaneous incremental increase per category resulted in the maximum sampling condition S10 which included the mean of the first 10 × 1 s sequences of category F, the mean of the first 10 × 2 s sequences of category E, and the overall mean of categories C and D and the single dataset of category A and B.

The statistical analysis was performed using IBM SPSS version 29 (IBM, Chicago, USA). Descriptive statistics were calculated for all COP data as well as for each sequence (S1–S10). The normal distribution of the obtained COP data was validated by the Shapiro–Wilk test.

The intra-class correlation coefficient (ICC) was used to assess the inter-observer reliability of all calculated COP parameters for each sequence (S1–S10). This assessment was based on the analyzed data between three observers (NA, MA, and CL). Additionally, the test-retest reliability of the analytical method was determined by an ICC analysis when comparing all three trials (M1–M3) for each dog by all observers to evaluate the consistency of COP data between trials (and thus also over time). Furthermore, the ICC was used to assess the reliability between different measurement durations (1–20 s) of each sequence (S1–S10). The ICC ranged from 1 (excellent correlation) to 0 (no correlation). An ICC > 0.8 was considered excellent correlation, 0.79 > ICC > 0.60 was considered very good correlation, 0.59 > ICC > 0.40 was considered good correlation, 0.39 > ICC > 0.20 was considered questionable, and an ICC < 0.20 was considered unacceptable (51). In this study, a robust correlation was considered to be achieved when an ICC ≥ 0.60, indicating a range from very good to excellent correlation (51).

To determine significant differences between measurement durations (1–20 s), a one-way analysis of variance (ANOVA) test was carried out, followed by a Bonferroni *post-hoc* test, employing a statistical significance level set at *p* < 0.05.

## 3 Results

Among the 12 dogs studied, seven dogs had incomplete categories, because they failed to stand still continuously for 20 s in at least one of the three trials (M1–M3). For example, achieving valid measurements without any head or tail movements was impossible for two observers in dog 8. As a result, one trial (M3) of this dog was excluded from further analysis. Table 1 displays the maximum number of analyzed seconds in each category for all incomplete measurements for each dog and observer.

Descriptive statistics (mean ± SD) are displayed in Table 2 for each sequence separately. While the average speed (AS) remains similar across all sequences, all other evaluated COP parameters increase with increasing measurement duration. This observation was confirmed by an ANOVA; all analyzed COP parameters (except for AS) showed a significant difference when the measurement durations were compared with each other (e.g., 1 vs. 2 s, 1 vs. 5 s, 1 vs. 10 s, etc.; for details including all *p*-values see Supplementary Table 2).

**Table 2:** Descriptive statistics (mean ± SD) of all center of pressure (COP) parameters within each sequence across various measurement durations.

		**All**	**S1**	**S2**	**S3**	**S4**	**S5**	**S6**	**S7**	**S8**	**S9**	**S10**
**Parameter**	**Time (s)**	**Mean** ±**SD**										
MLD (mm)	1	2.39 ± 0.69	2.33 ± 0.68	2.34 ± 0.70	2.32 ± 0.69	2.32 ± 0.69	2.33 ± 0.68	2.34 ± 0.67	2.34 ± 0.68	2.35 ± 0.68	2.35 ± 0.68	2.35 ± 0.67
	2	2.84 ± 0.81	2.80 ± 0.83	2.77 ± 0.81	2.76 ± 0.78	2.76 ± 0.77	2.77 ± 0.78	2.79 ± 0.82	2.80 ± 0.81	2.82 ± 0.81	2.83 ± 0.81	2.84 ± 0.81
	5	3.61 ± 1.14	3.68 ± 1.35	3.62 ± 1.21	3.59 ± 1.17	3.61 ± 1.14						
	10	4.60 ± 1.74	4.68 ± 1.90	4.60 ± 1.74							
	15	5.31 ± 2.31	5.31 ± 2.31									
	20	6.11 ± 2.80	6.11 ± 2.80									
CCD (mm)	1	6.73 ± 1.84	6.50 ± 1.54	6.39 ± 1.64	6.42 ± 1.72	6.50 ± 1.82	6.54 ± 1.80	6.55 ± 1.78	6.58 ± 1.77	6.60 ± 1.78	6.62 ± 1.80	6.63 ± 1.81
	2	7.69 ± 1.98	7.41 ± 1.74	7.51 ± 1.87	7.50 ± 1.83	7.56 ± 1.86	7.59 ± 1.88	7.60 ± 1.93	7.63 ± 1.93	7.64 ± 1.93	7.65 ± 1.95	7.69 ± 1.98
	5	9.29 ± 2.27	9.10 ± 2.15	9.17 ± 2.16	9.23 ± 2.20	9.29 ± 2.27						
	10	10.73 ± 2.75	10.83 ± 2.65	10.73 ± 2.75							
	15	11.98 ± 3.31	11.98 ± 3.31									
	20	12.94 ± 3.80	12.94 ± 3.80									
L (m)	1	0.13 ± 0.04	0.12 ± 0.03	0.12 ± 0.03	0.12 ± 0.03	0.12 ± 0.05	0.12 ± 0.05	0.12 ± 0.04	0.12 ± 0.04	0.12 ± 0.04	0.12 ± 0.04	0.12 ± 0.04
	2	0.25 ± 0.06	0.24 ± 0.05	0.24 ± 0.06	0.24 ± 0.06	0.24 ± 0.06	0.25 ± 0.06	0.25 ± 0.06	0.25 ± 0.06	0.25 ± 0.06	0.25 ± 0.06	0.25 ± 0.06
	5	0.63 ± 0.14	0.61 ± 0.14	0.62 ± 0.14	0.62 ± 0.14	0.63 ± 0.14						
	10	1.26 ± 0.27	1.24 ± 0.26	1.26 ± 0.27								
	15	1.86 ± 0.39	1.86 ± 0.39									
	20	2.55 ± 0.51	2.55 ± 0.51									
AS (mm/s)	1	125.32 ± 30.05	117.73 ± 26.13	119.49 ± 28.96	120.25 ± 28.92	121.74 ± 29.74	122.21 ± 29.29	122.84 ± 29.28	123.07 ± 29.15	123.18 ± 29.29	123.20 ± 29.81	123.26 ± 29.73
	2	125.40 ± 28.85	119.14 ± 26.08	120.53 ± 27.78	121.77 ± 27.84	122.47 ± 28.40	123.12 ± 28.69	123.80 ± 28.57	124.22 ± 28.45	124.71 ± 28.75	125.15 ± 28.89	125.40 ± 28.85
	5	125.26 ± 27.80	121.57 ± 27.17	123.01 ± 27.34	124.06 ± 27.41	125.26 ± 27.80						
	10	125.96 ± 26.76	123.67 ± 25.92	125.96 ± 26.76							
	15	124.23 ± 25.85	124.23 ± 25.85									
	20	127.37 ± 25.66	127.37 ± 25.66									
SS (mm^2^)	1	9.86 ± 4.95	9.25 ± 4.47	9.24 ± 4.60	9.27 ± 4.52	9.29 ± 4.53	9.39 ± 4.52	9.39 ± 4.55	9.41 ± 4.54	9.45 ± 4.53	9.51 ± 4.61	9.57 ± 4.65
	2	11.64 ± 6.63	11.01 ± 5.85	11.00 ± 5.81	11.03 ± 5.60	11.06 ± 5.50	11.19 ± 5.71	11.36 ± 6.89	11.38 ± 6.68	11.47 ± 6.65	11.52 ± 6.54	11.64 ± 6.63
	5	15.32 ± 9.34	15.60 ± 11.01	15.28 ± 9.76	15.12 ± 9.46	15.32 ± 9.34						
	10	20.93 ± 12.89	21.14 ± 12.26	20.93 ± 12.89							
	15	26.83 ± 17.91	26.83 ± 17.91									
	20	33.36 ± 22.06	33.36 ± 22.06									

MLD, mediolateral displacement; CCD, craniocaudal displacement; L, total length of COP; AS, average speed of COP; SS, support surface of COP; S, sequence.

### 3.1 Inter-observer reliability

The ICC of all evaluated COP parameters between the three observers reached values between 0.93 and 0.98 in all measurement durations and was therefore classified as excellent inter-observer reliability.

### 3.2 Test-retest reliability

Each observer chose a 20 s length (or in some cases the maximum duration achievable, which is 15 s) within each trial independently. As a result, no identical starting point was chosen for the beginning of a trial. The difference in starting points between the three observers varied from 0.01 to 33.44 s. In 74% of the trials, the difference between the selected starting points was between 0.01 and 5.99 s. For details see Supplementary Table 3.

The ICC between the three trials (M1–M3) varied tremendously and depended on the measurement duration and the number of analyzed sequences (Table 3). The lowest ICC was observed when evaluating MLD when comparing 1 sequence (S1) lasting 1 s (ICC 0.18). The highest ICC was recorded for CCD when comparing 1 sequence (S1) lasting 20 s (ICC 0.82). Despite this single high ICC of CCD in the 20 s duration measurement, L and AS never reached the threshold >0.6, indicating a low test-retest reliability of these two parameters over the 20 s period.

**Table 3:** Test-retest reliability between three measurements (M1–M3).

**Parameter**	**Time (s)**	**S1**	**S2**	**S3**	**S4**	**S5**	**S6**	**S7**	**S8**	**S9**	**S10**
MLD	1	0.18	0.55	**0.68**							
CCD		0.41	0.52	0.57	0.53	0.54	0.51	0.53	0.54	0.52	0.52
L		0.43	0.59	0.57	0.4	0.42	0.5	0.52	0.58	0.59	**0.62**
AS		0.45	0.55	0.56	0.52	0.54	0.55	0.59	**0.61**		
SS		0.43	0.56	**0.61**							
MLD	2	0.56	**0.62**								
CCD		0.58	**0.62**								
L		0.57	0.53	0.52	0.53	0.53	0.52	0.53	0.54	0.58	0.58
AS		0.57	0.54	0.52	0.52	0.53	0.51	0.53	0.57	0.58	0.57
SS		0.55	**0.68**								
MLD	5	**0.65**									
CCD		0.50	**0.65**								
L		**0.67**									
AS		**0.67**									
SS		0.55	**0.64**								
MLD	10	**0.68**									
CCD		**0.78**									
L		**0.60**									
AS		**0.60**									
SS		**0.73**									
MLD	15	**0.60**									
CCD		**0.75**									
L		**0.61**									
AS		**0.61**									
SS		**0.61**									
MLD	20	**0.72**									
CCD		**0.82**									
L		0.55									
AS		0.55									
SS		**0.76**									

The minimum number of sequences (S1–S10) per measurement duration needed to reach an ICC ≥ 0.60 (indicated as numbers written in bold) across all three trials.

When investigating the minimum number of sequences needed for each measurement duration, it was observed that only in the 5, 10, and 15 s duration all COP parameters reached the threshold (ICC ≥ 0.60). However, for the CCD and SS to become reliable in the 5 s duration two sequences (2 times 5 s) were needed, whereas in the 10 and 15 s duration, a single sequence was sufficient. In all other measurement durations, at least one parameter (e.g., CCD in the 1 s measurement), and in some cases, two parameters (e.g., L and AS in the 2 and 20 s measurements) did not reach the threshold in any of the analyzed sequences (S1–S10, see Table 3).

Table 4 illustrates the minimum number of sequences required for each parameter to meet the defined threshold. In summary, it was observed that at least one of the COP parameters in the 1, 2, and 20 s durations did not reach the threshold. In the 5 s durations one sequence was sufficient for MLD, L, and AS, while for the CCD and SS, two sequences had to be used. Across all COP parameters, one sequence was sufficient for the 10 and 15 s durations.

**Table 4:** Illustration of the minimum number of sequences needed to reach the threshold (ICC ≥ 0.60).

**Duration of measurement (s)**	**COP parameter**	**Number of required sequences**
1	MLD	3
	CCD	n.r.^*^
	L	10
	AS	8
	SS	3
2	MLD	2
	CCD	2
	L	n.r.^*^
	AS	n.r.^*^
	SS	2
5	MLD	1
	CCD	2
	L	1
	AS	1
	SS	2
10	MLD	1
	CCD	1
	L	1
	AS	1
	SS	1
15	MLD	1
	CCD	1
	L	1
	AS	1
	SS	1
20	MLD	1
	CCD	1
	L	n.r.^*^
	AS	n.r.^*^
	SS	1

^*^n.r., never reached the threshold.

### 3.3 Correlation between measurement durations and sequences

In the final step, we investigated the correlation between measurement durations and all sequences (S1–S10) with each other (e.g., 1 s, S1 vs. 2 s, S1; 1 s, S1 vs. 5 s, S1; 1 s, S1 vs. 10 s, S1; for details see Table 5). In general, the ICC tends to decrease the more the measurement durations deviate from one another. A very good to excellent ICC was consistently achieved (for all 5 COP parameters) whenever the measurement duration doubled at most (e.g., 1 vs. 2 s; 5 vs. 10 s; 10 vs. 20 s, but not 1 vs. 5 s). When analyzing the number of sequences (S1–S10), single sequences (S1) already achieved a very good and excellent ICC's in 1 s S1 vs. 2 s S1 (ICC 0.74–0.95), 5 s S1 vs. 10 s S1 (ICC 0.72–0.94), 10 s S1 vs. 15 s S1 (ICC 0.71–0.90), 15 s S1 vs. 20 s S1 (ICC 0.87–0.99), and 10 s S1 vs. 20 s S1 (ICC 0.77–0.96).

**Table 5:** Illustration of the ICC between the individual measurement durations for each COP parameter.

**Parameter**	**Time (s)**	**S1**	**S2**	**S3**	**S4**	**S5**	**S6**	**S7**	**S8**	**S9**	**S10**
MLD	1 vs.	2	**0.77**									
CCD			**0.85**									
L			**0.74**									
AS			**0.95**									
SS			**0.78**									
MLD		5	0.27	**0.66**								
CCD			0.46	**0.82**								
L			0.28	0.51	0.46	0.47	0.45	0.45	0.46	0.45	0.46	0.45
AS			**0.76**									
SS			0.29	**0.66**								
MLD		10	0.27	0.45	0.46	0.45	0.44	0.43	0.43	0.43	0.44	0.44
CCD			0.47	0.60	**0.62**							
L			0.16	0.19	0.19	0.24	0.22	0.22	0.20	0.22	0.22	0.22
AS			**0.80**									
SS			0.35	0.54	0.54	0.55	0.55	0.54	0.53	0.52	0.52	0.52
MLD		15	0.49	0.28	0.27	0.25	0.24	0.23	0.22	0.22	0.23	0.23
CCD			0.39	**0.61**								
L			0.10	0.22	0.22	0.27	0.26	0.26	0.26	0.25	0.26	0.25
AS			**0.74**									
SS			0.18	0.28	0.27	0.28	0.27	0.27	0.26	0.26	0.26	0.26
MLD		20	0.11	0.19	0.18	0.16	0.15	0.13	0.11	0.10	0.11	0.11
CCD			0.35	0.58	0.58	**0.61**						
L			0.07	0.17	0.16	0.22	0.21	0.20	0.20	0.20	0.20	0.19
AS			**0.69**									
SS			0.17	0.26	0.26	0.27	0.27	0.27	0.26	0.24	0.23	0.23
MLD		5	0.54	**0.78**								
CCD	2 vs.		**0.63**									
L			0.57	**0.77**								
AS			**0.85**									
SS			0.52	**0.80**								
MLD		10	0.51	0.55	0.55	0.56	0.57	0.60	**0.62**			
CCD			**0.61**									
L			0.33	0.35	0.35	0.37	0.36	0.37	0.38	0.42	0.46	0.48
AS			**0.87**									
SS			**0.60**									
MLD		15	0.37	0.41	0.39	0.39	0.39	0.43	0.42	0.41	0.39	0.37
CCD			0.50	**0.70**								
L			0.21	0.39	0.40	0.41	0.42	0.41	0.41	0.42	0.42	0.43
AS			**0.81**									
SS			0.31	0.42	0.40	0.41	0.41	0.53	0.52	0.51	0.49	0.49
MLD		20	0.25	0.24	0.24	0.25	0.25	0.28	0.28	0.28	0.28	0.29
CCD			0.46	**0.65**								
L			0.16	0.31	0.31	0.32	0.33	0.33	0.33	0.33	0.33	0.33
AS			**0.78**									
SS			0.28	0.36	0.36	0.37	0.35	0.43	0.43	0.43	0.42	0.43
MLD		10	**0.75**									
CCD	5 vs.		**0.72**									
L			**0.77**									
AS			**0.94**									
SS			**0.79**									
MLD		15	0.54	**0.70**								
CCD			**0.66**									
L			0.58	**0.74**								
AS			**0.93**									
SS			0.44	**0.67**								
MLD		20	0.40	0.59	0.59	0.58	0.58	0.58	0.58	0.58	0.58	0.58
CCD			**0.60**									
L			0.46	**0.63**								
AS			**0.92**									
SS			0.44	**0.62**								
MLD	10 vs.	15	**0.84**									
CCD			**0.88**									
L			**0.90**									
AS			**0.97**									
SS			**0.71**									
MLD		20	**0.77**									
CCD			**0.86**									
L			**0.77**									
AS			**0.96**									
SS			**0.66**									
MLD	15 vs.	20	**0.89**									
CCD			**0.94**									
L			**0.95**									
AS			**0.99**									
SS			**0.87**									

The table presents the minimum number of sequences required to achieve the threshold (ICC ≥ 0.60, indicated as numbers written in bold) across varying measurement durations.

However, when larger differences in measurement duration were assessed, the number of COP parameters reaching the threshold continuously decreased. For instance, when comparing 1 s measurements with 10, 15, and 20 s measurements, only two COP parameters reached the threshold (CCD and AS) whereas three parameters never reached the threshold in any of the sequences. It is noteworthy that AS and CCD consistently met the required ICC when comparing all measurement durations with each other in one of the analyzed sequences.

## 4 Discussion

In this study, we assessed the inter-observer reliability and test-retest reliability of body COP parameters of healthy young to middle-aged dogs during quiet standing on a pressure plate. Moreover, our objective was to determine the minimum number of trials needed and the shortest measurement duration necessary to obtain reliable and comparable COP data.

The study demonstrated excellent inter-observer reliability for all COP parameters, demonstrating the reproducibility of the analyzed time period within each trial across different observers. Additionally, according to test-retest reliability from three sets of trials, achieving a very good to excellent correlation requires a minimum of two 5 s measurements or a single 10 or 15 s measurement.

Test-retest reliability serves as a measure to evaluate the consistency or stability of a measure or test over time, assessing if repeated assessments of the same individuals yield similar results under consistent conditions. When evaluating test-retest reliability across the three repeated trials (M1 vs. M2 vs. M3) for each dog, we were able to demonstrate that only the 5, 10, and 15 s measurement duration met the predetermined threshold (ICC ≥ 0.60) in all evaluated COP parameters. In all other measurement durations (1, 2, and 20 s), 1 or 2 COP parameters failed to meet the ICC criterion in all analyzed sequences. The required minimum number of sequences was two sequences for the 5 s and one sequence for the 10 and 15 s duration, respectively.

Finding the shortest measurement duration to gain reliable COP parameters is not only in line with the 3R principle, emphasizing the reduction and refinement of animal trials (47) but also holds crucial practical significance. Standing still for long enough is repeatedly stated to be one of the most challenging tasks in static posturography literature (25). In our own experience, standing motionless is especially challenging for young untrained dogs and elderly dogs facing certain clinical conditions such as neurodegenerative diseases. Finding the shortest needed measurement duration would also aid scientists when evaluating different species, particularly wild animals such as elephants (41) and flamingos (38) that might not remain still for extended periods. To the best of our knowledge, no research article has yet attempted to identify the shortest reproducible and repeatable measurement duration for COP parameters in veterinary medicine. Indeed, the veterinary literature documents a wide range of measurement durations and repetitions when evaluating COP data in static posturography. While the majority of these articles focus on dogs (13, 15, 20, 25, 34–37) and horses (2, 39, 42–45, 52), others also explore species like cats (53), elephants (41), flamingos (38), and rats (54). The longest analyzed duration among these articles was 30 s in dogs (34) followed by 20 s in dogs (13, 15, 36) and ponies (2) and 15 s in horses (52); all these aforementioned papers used three repetitions. However, in various articles, even shorter periods were analyzed. In some articles, 10 s measurements were repeated from 3 (20, 35) to 5 (39, 42) times, whereas, in other literature even shorter durations such as 5 s (46), 3 s (23), or 1 s (25, 41) were measured.

Our findings indicate that two 5 s measurements or one single 10 s measurement are the shortest measurement durations with reproducible results that yield a very good ICC. Thus, based on our results we strongly encourage future studies to implement a more standardized and verified protocol, not only to reduce the impact and potential welfare implications on the study participants but also to make study results more comparable within-, and potentially between-species.

Despite these highly relevant results it needs to be pointed out that, based on the ANOVA results statistically significant differences were found among all measurement durations for all COP parameters (except AS). This means that despite very good to excellent correlations, COP parameters that have been assessed over a 5 s period cannot be directly compared to COP parameters assessed over 10 s within the same animal and across the study participants. Thus, when analyzing COP parameters, we suggest using identical measurement durations (e.g., two times 5 s or one time 10 s) for all dogs participating in the same study.

By employing different analytical methods for COP data in static posturography within this study, significantly different outcomes were obtained. Most surprisingly this included the 20 s measurement duration, which is one the most often used protocol (analyzing three times 20 s) published (13, 15, 36). Despite high correlations across the three trials (M1–M3) for CCD, MLD, and SS (0.72–0.82), L and AS failed to meet the ICC criterion, thus questioning this protocol to be used in the future.

As already mentioned above, measurement duration is a critical aspect when working with dogs. Certain COP parameters might already incorporate important clinical information within a short time span. For example, based on our results, MLD, CCD, and SS achieve ICC ≥ 0.6, when using at least two 2 s sequences, whereas L and AS did not reach the threshold even when we used 10 2 s sequences. This finding might be relevant for elderly dogs and animals with certain pathological conditions such as neurodegenerative or musculoskeletal diseases. These impaired animals might not be able to stand still for long periods (e.g., two times 5 s) so that the full range of COP parameters cannot be evaluated. Therefore, further studies need to be conducted to determine certain COP parameters capture clinically relevant balance and body sway aspects in short timespans in animals that are not able to stand still for longer periods.

This study evaluated an extensive panel of COP parameters. Those conventional (linear) COP parameters are indicators of postural stability (55, 56). Based on our results, COP parameters might have been affected by measurement duration. It is noteworthy that the shortest (1 s) and the longest measurement duration (20 s) had the least reliable results. To the best of our knowledge, this has not been published in veterinary medicine before. However, in recent years, non-linear methods such as sample entropy and approximate entropy have been investigated in human medicine (57, 58). These non-linear algorithms evaluate the randomness of data series and analyze the non-linearity in postural sway dynamics by assessing irregularities present in the COP's time series data (57, 58). Future studies should explore those additional analytical methods to focus on this phenomenon in more detail.

In this study, one of the three observers generated the trial data for a dog. However, to analyze the COP parameters, each observer selected a starting point within each trial of all dogs individually. Each observer underwent practical training involving various dogs and different test conditions beforehand. This included finding the ideal 20 s measurement duration within each trial (M1, M2, and M3), during which dogs stood still for further analysis. In addition, they were specifically trained in the analysis software and adhered to a standard operating protocol. Despite differing starting points (difference of 0.01–33.44 s) an excellent ICC (>0.93) was achieved. Consequently, it can be inferred that they possessed equal levels of expertise in data acquisition, which was confirmed by the excellent inter-observer reliability.

A limitation of this study was the absence of a third analysis (M3) for one of the included dogs. Unlike the other dogs, in this particular one achieving valid sequences without any head and tail movements from two of the observers was impossible. As a result, one trial (M3) was excluded. Seven dogs exhibited incomplete 20 s sequences, leading to the selection of the longest valid period for analysis in these dogs (e.g., 15 s long period). It needs to be pointed out that only 50% of dogs were able to stand still for at least 30 consecutive seconds. Another limitation of this study is the utilization of a small cohort of healthy dogs. Although the low sample size may not affect the agreement (ICC) between observers, it affects the size of the analyzed dataset (ANOVA). This is why we suggest incorporating a more diverse range of dogs with varying morphologies and anthropometric parameters (body weight and height) (25, 37), as well as aged dogs and those with different orthopedic and neurological conditions (13, 15, 20). This will help to offer additional insights into the parameters under investigation. Furthermore, exploring the influence of muscular fatigue when standing over prolonged periods on COP data could present a promising avenue for future research.

## 5 Conclusion

In conclusion, these findings suggest that conducting one 10 s or two 5 s measurements yields optimal results for the assessment of postural balance in dogs when analyzing conventional COP data during static posturography. Further exploration of physiological and pathological factors and the impact of more subtle movements during static posturography such as breathing frequency could enhance this measurement approach. Establishing a standardized methodology based on these insights would enable researchers to draw more robust conclusions from existing studies and facilitate easier comparisons between published results and their research findings.

## Data availability statement

The datasets presented in this study can be found in online repositories. The names of the repository/repositories and accession number(s) can be found in the article/Supplementary material.

## Ethics statement

The animal studies were approved by the Ethics and Animal Welfare Committee of the University of Veterinary Medicine, Vienna in accordance with the University's guidelines for Good Scientific Practice (ETK-148/10/2021). The studies were conducted in accordance with the local legislation and institutional requirements. Written informed consent was obtained from the owners for the participation of their animals in this study.

## Author contributions

MA: Conceptualization, Data curation, Formal analysis, Investigation, Methodology, Validation, Visualization, Writing – original draft, Writing – review & editing. NA: Conceptualization, Data curation, Formal analysis, Investigation, Methodology, Validation, Visualization, Writing – original draft, Writing – review & editing. CL: Conceptualization, Data curation, Formal analysis, Investigation, Methodology, Validation, Visualization, Writing – original draft, Writing – review & editing. CP: Conceptualization, Data curation, Formal analysis, Investigation, Methodology, Validation, Visualization, Writing – original draft, Writing – review & editing. AT: Formal analysis, Methodology, Validation, Writing – review & editing. BB: Conceptualization, Data curation, Formal analysis, Funding acquisition, Investigation, Methodology, Project administration, Resources, Supervision, Validation, Visualization, Writing – original draft, Writing – review & editing.
