# Supplementary material for: A validation study to analyze the reliability of center of pressure data in static posturography in dogs
Source: Front Vet Sci. 2024 Mar 14;11:1353824. doi: 10.3389/fvets.2024.1353824 (PMC10979645; doi:10.3389/fvets.2024.1353824)
Supplement: Supplementary file 1 [file Data_Sheet_1.PDF]

Table S1: Overview of the cited literature based on the trial number, trial duration, species, and investigated parameters.

Reference in Paper	Publication	Number of Trials	Duration of each Trial (s)	Species	Investigated Parameters
2	Pitti et al. 2018	3	20	Horse	COP
13	López et al. 2019	3	20	Dog	COP
15	Carrillo et al. 2018	3	20	Dog	COP
20	Manera et al. 2017	3	10	Dog	COP
23	Linder et al. 2021	6	n.a.	Dog	GRF
24	Clough et al. 2018	15	n.a.	Dog	Companion stance analyzer
25	Mondino et al. 2022	7	1	Dog	COP
26	Heffernan et al. 2018	3	10	Dog	GRF
27	Davila et al. 2013	2	10	Dog	GRF
28	Horstman et al. 2004	5	3	Dog	GRF
29	Lascelles et al. 2006	5	5	Dog	GRF
30	Lee et al. 1999	3	10	Dog	GRF
31	Phelps et al. 2007	4	6	Dog	Static quadruped load distribution
32	Seibert et al. 2012	1	13	Dog	GRF
34	Lee et al. 2019	3	30	Dog	COP
35	Lee et al. 2019	3	10	Dog	COP
36	Lutonsky et al. 2023	3	20	Dog	COP
37	Shaheen et al. 2023	n.a.	10	Dog	COP
38	Chang et al. 2017	n.a.	n.a.	Flamingo	COP
39	Clayton et al. 2003	5	10	Horse	COP
40	Clayton et al. 2013	1	15	Horse	COP
41	Dewolf et al. 2021	1	4.4	Elephant and dog	COP
42	Ellis and King 2020	10	5	Horse	COP
43	Gomes-Costa et al. 2015	n.a.	n.a.	Horse	COP
44	Moorman et al. 2017	10	n.a.	Horse	COP
45	Nauwelaerts et al. 2013	8	3	Horse	COP
46	Schleining et al. 2011	5	1	Horse	COP
52	Clayton et al. 2014	15	3	Horse	COP

n.a.: not available

Figure S1: Illustration of the camera setup utilized in the study.

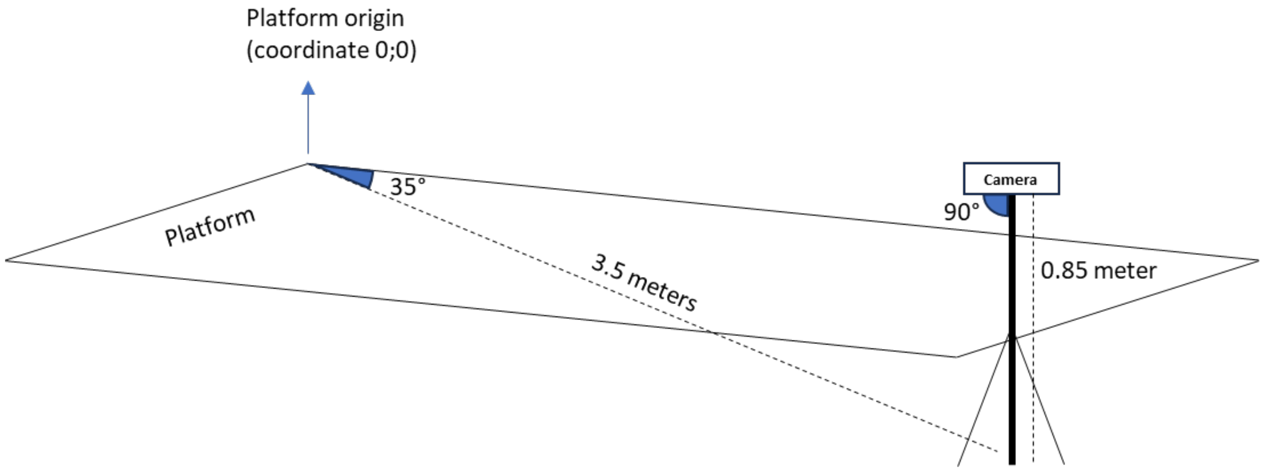

Table S2: The results of the ANOVA and Bonferroni post hoc test with a significance level of 0.05 for all measurement durations.

COP parameter	Measurement duration (s)	Measurement duration (s)	Mean difference	Standard error	p-value	95% Confidence Intervall	
MLD (mm)	1	2	-0.44	0.04	0.00	-0.56	-0.33
		5	-1.22	0.06	0.00	-1.38	-1.06
		10	-2.21	0.08	0.00	-2.44	-1.98
		15	-2.92	0.10	0.00	-3.23	-2.61
		20	-3.72	0.11	0.00	-4.05	-3.39
	2	1	0.44	0.04	0.00	0.33	0.56
		5	-0.78	0.06	0.00	-0.95	-0.60
		10	-1.76	0.08	0.00	-2.00	-1.53
		15	-2.48	0.11	0.00	-2.79	-2.16
		20	-3.28	0.11	0.00	-3.61	-2.94
	5	1	1.22	0.06	0.00	1.06	1.38
		2	0.78	0.06	0.00	0.60	0.95
		10	-0.99	0.09	0.00	-1.25	-0.73
		15	-1.70	0.11	0.00	-2.04	-1.37
		20	-2.50	0.12	0.00	-2.85	-2.15
	10	1	2.21	0.08	0.00	1.98	2.44
		2	1.76	0.08	0.00	1.53	2.00
		5	0.99	0.09	0.00	0.73	1.25
		15	-0.71	0.13	0.00	-1.08	-0.34
		20	-1.51	0.13	0.00	-1.90	-1.12
	15	1	2.92	0.10	0.00	2.61	3.23
		2	2.48	0.11	0.00	2.16	2.79
		5	1.70	0.11	0.00	1.37	2.04
		10	0.71	0.13	0.00	0.34	1.08
		20	-0.80	0.15	0.00	-1.24	-0.36
	20	1	3.72	0.11	0.00	3.39	4.05
		2	3.28	0.11	0.00	2.94	3.61
		5	2.50	0.12	0.00	2.15	2.85
		10	1.51	0.13	0.00	1.12	1.90
		15	0.80	0.15	0.00	0.36	1.24
CCD (mm)	1	2	-0.96	0.08	0.00	-1.20	-0.72
		5	-2.56	0.11	0.00	-2.90	-2.23
		10	-4.00	0.16	0.00	-4.47	-3.54
		15	-5.26	0.22	0.00	-5.89	-4.62
		20	-6.21	0.23	0.00	-6.89	-5.54
	2	1	0.96	0.08	0.00	0.72	1.20
		5	-1.60	0.12	0.00	-1.97	-1.24

		10	-3.05	0.17	0.00	-3.53	-2.56
		15	-4.30	0.22	0.00	-4.94	-3.65
		20	-5.26	0.23	0.00	-5.94	-4.57
	5	1	2.56	0.11	0.00	2.23	2.90
		2	1.60	0.12	0.00	1.24	1.97
		10	-1.44	0.18	0.00	-1.98	-0.90
		15	-2.69	0.24	0.00	-3.38	-2.00
		20	-3.65	0.25	0.00	-4.38	-2.92
	10	1	4.00	0.16	0.00	3.54	4.47
		2	3.05	0.17	0.00	2.56	3.53
		5	1.44	0.18	0.00	0.90	1.98
		15	-1.25	0.26	0.00	-2.01	-0.49
		20	-2.21	0.27	0.00	-3.00	-1.41
	15	1	5.26	0.22	0.00	4.62	5.89
		2	4.30	0.22	0.00	3.65	4.94
		5	2.69	0.24	0.00	2.00	3.38
		10	1.25	0.26	0.00	0.49	2.01
		20	-0.96	0.31	0.03	-1.86	-0.06
	20	1	6.21	0.23	0.00	5.54	6.89
		2	5.26	0.23	0.00	4.57	5.94
		5	3.65	0.25	0.00	2.92	4.38
		10	2.21	0.27	0.00	1.41	3.00
		15	0.96	0.31	0.03	0.06	1.86
L (m)	1	2	-0.13	0.01	0.00	-0.14	-0.11
		5	-0.50	0.01	0.00	-0.52	-0.48
		10	-1.13	0.01	0.00	-1.16	-1.10
		15	-1.74	0.01	0.00	-1.78	-1.70
		20	-2.42	0.01	0.00	-2.46	-2.38
	2	1	0.13	0.01	0.00	0.11	0.14
		5	-0.38	0.01	0.00	-0.40	-0.35
		10	-1.01	0.01	0.00	-1.04	-0.98
		15	-1.61	0.01	0.00	-1.65	-1.57
		20	-2.30	0.01	0.00	-2.34	-2.25
	5	1	0.50	0.01	0.00	0.48	0.52
		2	0.38	0.01	0.00	0.35	0.40
		10	-0.63	0.01	0.00	-0.67	-0.60
		15	-1.24	0.01	0.00	-1.28	-1.19
		20	-1.92	0.02	0.00	-1.97	-1.88
	10	1	1.13	0.01	0.00	1.10	1.16
		2	1.01	0.01	0.00	0.98	1.04
		5	0.63	0.01	0.00	0.60	0.67
		15	-0.60	0.02	0.00	-0.65	-0.56

		20	-1.29	0.02	0.00	-1.34	-1.24
	15	1	1.74	0.01	0.00	1.70	1.78
		2	1.61	0.01	0.00	1.57	1.65
		5	1.24	0.01	0.00	1.19	1.28
		10	0.60	0.02	0.00	0.56	0.65
		20	-0.68	0.02	0.00	-0.74	-0.63
	20	1	2.42	0.01	0.00	2.38	2.46
		2	2.30	0.01	0.00	2.25	2.34
		5	1.92	0.02	0.00	1.88	1.97
		10	1.29	0.02	0.00	1.24	1.34
		15	0.68	0.02	0.00	0.63	0.74
AS (mm/s)	1	2	-0.07	1.13	1.00	-3.40	3.25
		5	0.06	1.60	1.00	-4.64	4.76
		10	-0.64	2.21	1.00	-7.12	5.85
		15	1.09	3.00	1.00	-7.72	9.91
		20	-2.05	3.19	1.00	-11.42	7.33
	2	1	0.07	1.13	1.00	-3.25	3.40
		5	0.14	1.73	1.00	-4.95	5.22
		10	-0.56	2.30	1.00	-7.33	6.20
		15	1.17	3.07	1.00	-7.85	10.19
		20	-1.97	3.26	1.00	-11.54	7.60
	5	1	-0.06	1.60	1.00	-4.76	4.64
		2	-0.14	1.73	1.00	-5.22	4.95
		10	-0.70	2.57	1.00	-8.24	6.84
		15	1.03	3.27	1.00	-8.58	10.65
		20	-2.11	3.45	1.00	-12.24	8.02
	10	1	0.64	2.21	1.00	-5.85	7.12
		2	0.56	2.30	1.00	-6.20	7.33
		5	0.70	2.57	1.00	-6.84	8.24
		15	1.73	3.61	1.00	-8.87	12.33
		20	-1.41	3.77	1.00	-12.48	9.66
	15	1	-1.09	3.00	1.00	-9.91	7.72
		2	-1.17	3.07	1.00	-10.19	7.85
		5	-1.03	3.27	1.00	-10.65	8.58
		10	-1.73	3.61	1.00	-12.33	8.87
		20	-3.14	4.28	1.00	-15.72	9.44
	20	1	2.05	3.19	1.00	-7.33	11.42
		2	1.97	3.26	1.00	-7.60	11.54
		5	2.11	3.45	1.00	-8.02	12.24
		10	1.41	3.77	1.00	-9.66	12.48
		15	3.14	4.28	1.00	-9.44	15.72
SS (mm^2)	1	2	-1.78	0.30	0.00	-2.68	-0.88

	5	-5.47	0.43	0.00	-6.73	-4.20
	10	-11.07	0.59	0.00	-12.82	-9.32
	15	-16.97	0.81	0.00	-19.35	-14.60
	20	-23.50	0.86	0.00	-26.03	-20.97
2	1	1.78	0.30	0.00	0.88	2.68
	5	-3.69	0.47	0.00	-5.05	-2.32
	10	-9.29	0.62	0.00	-11.11	-7.47
	15	-15.19	0.83	0.00	-17.62	-12.76
	20	-21.72	0.88	0.00	-24.30	-19.14
5	1	5.47	0.43	0.00	4.20	6.73
	2	3.69	0.47	0.00	2.32	5.05
	10	-5.60	0.69	0.00	-7.63	-3.57
	15	-11.51	0.88	0.00	-14.10	-8.92
	20	-18.03	0.93	0.00	-20.77	-15.30
10	1	11.07	0.59	0.00	9.32	12.82
	2	9.29	0.62	0.00	7.47	11.11
	5	5.60	0.69	0.00	3.57	7.63
	15	-5.90	0.97	0.00	-8.76	-3.05
	20	-12.43	1.02	0.00	-15.41	-9.45
15	1	16.97	0.81	0.00	14.60	19.35
	2	15.19	0.83	0.00	12.76	17.62
	5	11.51	0.88	0.00	8.92	14.10
	10	5.90	0.97	0.00	3.05	8.76
	20	-6.53	1.15	0.00	-9.92	-3.14
20	1	23.50	0.86	0.00	20.97	26.03
	2	21.72	0.88	0.00	19.14	24.30
	5	18.03	0.93	0.00	15.30	20.77
	10	12.43	1.02	0.00	9.45	15.41
	15	6.53	1.15	0.00	3.14	9.92

MLD: mediolateral displacement; CCD: craniocaudal displacement; L: total length of COP; AS: average speed of COP; SS: support surface of COP

Table S3: The difference in selected starting points chosen by three observers.

Difference (s)	Percent (%)
0s to 1.99s	39
2s to 3.99s	24
4s to 5.99	11
6s to 7.99s	5
8s to 9.99s	9
10s to 11.99s	5
12s and higher	7
